# Supplementary material for: Phenotypic and Genetic Study of the Presence of Hair Whorls in Pura Raza Español Horses
Source: Animals (Basel). 2023 Sep 16;13(18):2943. doi: 10.3390/ani13182943 (PMC10525084; doi:10.3390/ani13182943)
Supplement: Supplementary file 1 [file animals-13-02943-s001.zip › animals-2480421-supplementary.pdf]

Table S1. Generalized Non-Linear Model (GLZ) results using a binomial logit model depending on different risk factors and incidence rate percentage according to hair circular and linear whorl positions.

			Gender (N)			Inbreeding coefficient				Coat color					Birth period			
		Position	p-value	♂ (n:15,781)	♀ (n:27,616)	p-value	<0.125 (n:39,171)	0.125-0.1875 (n:2,954)	>0.1875 (n:1,272)	p-value	Bay (n:17,015)	Chestnut (n:3,504)	Black (n:5,443)	Grey (n:17,435)	p-value	≤2001 (n:634)	2002-2011 (n:12,741)	≥2012 (n:30,022)
Head	circular whorls	Top right	0.782	2.28	2.29	0.055	2.32	2.27	1.34	0.029	2.51	2.60	2.11	2.07	0.062	1.58	2.07	2.40
		Top center	0.449	25.21	24.65	0.753	24.88	24.48	24.92	<0.001	24.44	24.94	22.38	26.01	<0.001	15.14	22.20	26.18
		Top left	0.903	3.99	3.98	0.236	4.01	4.06	3.07	0.188	4.24	3.85	3.91	3.77	0.020	2.05	3.92	4.05
		Between eyes	<0.001	55.54	52.98	0.009	53.81	53.56	57.94	0.015	53.78	52.85	56.07	53.58	<0.001	42.59	57.30	52.71
		Low right	<0.001	45.34	51.69	0.880	49.27	50.71	49.61	<0.001	46.40	40.81	46.46	54.92	<0.001	10.10	40.20	54.14
		Low center	<0.001	16.87	19.80	0.827	18.80	18.08	18.32	<0.001	19.66	19.04	18.81	17.75	<0.001	13.25	18.30	19.04
		Low left	<0.001	47.22	53.79	0.511	51.21	53.22	53.07	<0.001	48.20	42.47	48.10	57.36	<0.001	10.10	41.60	56.44
	linear whorls	Top right	0.609	0.09	0.09	0.387	0.09	0.03	0.16	0.608	0.09	0.09	0.15	0.08	0.064	0.00	0.06	0.11
		Top center	<0.001	1.18	1.56	0.575	1.41	1.46	1.81	0.031	1.32	1.34	1.16	1.63	0.074	0.79	1.30	1.49
		Top left	0.605	0.16	0.19	0.818	0.18	0.24	0.16	0.106	0.17	0.11	0.09	0.23	0.098	0.00	0.17	0.19
		Between eyes	0.647	2.60	2.70	0.007	2.61	2.88	3.70	<0.001	2.41	1.74	2.44	3.17	0.108	42.50	57.30	52.71
		Low right	0.821	3.40	3.49	0.922	3.45	3.52	3.70	<0.001	3.03	3.23	3.51	3.91	<0.001	2.84	5.54	2.59
		Low center	<0.001	2.77	4.10	0.046	3.54	4.06	4.87	<0.001	3.52	2.34	3.05	4.14	<0.001	2.68	3.03	3.88
		Low left	0.541	3.55	3.57	0.209	3.51	3.89	4.40	<0.001	3.12	3.31	3.51	4.06	<0.001	2.84	5.60	2.72
Body-neck	Circular whorls	Right	<0.001	88.61	89.54	<0.001	88.99	90.69	92.37	<0.001	88.53	89.50	87.40	90.36	<0.001	56.78	89.63	89.70
		Center	0.534	86.49	85.77	0.091	85.97	85.91	87.97	<0.001	85.42	87.53	84.97	86.65	<0.001	51.26	83.85	87.69
		Left	<0.001	88.66	89.67	<0.001	89.08	90.99	92.37	<0.001	88.50	89.36	87.54	90.63	<0.001	57.10	89.65	89.84
	linear whorls	Right	0.436	43.00	43.85	<0.001	42.99	48.51	50.00	<0.001	40.91	37.41	38.18	49.09	<0.001	29.02	39.13	45.77
		Center	0.002	57.02	55.50	<0.001	55.67	58.70	61.64	<0.001	55.35	55.79	53.63	57.55	<0.001	42.27	56.71	56.06
		Left	0.207	43.03	43.87	<0.001	42.98	48.44	50.16	<0.001	40.86	37.41	38.03	49.17	<0.001	29.18	39.02	45.80
Limbs	circualr whorls	Right forelimb	<0.001	2.37	1.17	0.014	1.62	1.76	0.71	0.080	1.79	1.28	1.60	1.49	0.009	0.63	1.81	1.54
		Left forelimb	<0.001	2.17	1.17	0.082	1.55	1.69	0.87	0.668	1.60	1.34	1.53	1.51	0.081	1.26	1.71	1.47
		Right hindlimb	<0.001	9.08	8.21	0.050	8.47	9.85	7.15	<0.001	8.00	7.39	7.62	9.54	<0.001	3.94	11.90	7.19
		Left hindlimb	<0.001	9.24	8.20	0.025	8.54	9.68	7.23	<0.001	8.00	7.65	7.81	9.57	<0.001	3.94	12.03	7.21
	linear whorls	Right forelimb	<0.001	0.91	0.46	0.397	0.62	0.75	0.393	0.432	0.59	0.63	0.81	0.6	<0.001	0.47	0.90	0.51
		Left forelimb	<0.001	0.83	0.43	0.237	0.56	0.81	0.47	0.873	0.55	0.57	0.64	0.59	0.002	0.79	0.75	0.50

	Right hindlimb	0.002	7.52	6.93	0.050	7.10	8.23	6.13	<0.001	6.36	6.08	6.12	8.44	<0.001	3.31	10.01	6.01
	Left hindlimb	0.001	7.61	6.98	0.087	7.17	8.09	6.21	<0.001	6.39	6.16	6.41	8.46	<0.001	2.10	10.18	6.04

Table S2. Generalized Non-Linear Model (GLZ) results using a multinomial logit model depending on different risk factors and incidence rate percentage according to the number of circular and linear hair whorls.

			Gender			Inbreeding coefficient				Coat color					Birth period			
			GLZ	♂	♀	GLZ	<0.125	0.125-0.1875	>0.1875	GLZ	Bay	Chestnut	Black	Grey	GLZ	≤2001	2002-2011	≥2012
		Number	p-value	(n:15,781)	(n:27,616)	p-value	(n:39,171)	(n:2,954)	(n:1,272)	p-value	(n:17,015)	(n:3,504)	(n:5,443)	(n:17,435)	p-value	(n:634)	(n:12,741)	(n:30,022)
Head	circualr whorls	0	<0.001	4.17	4.22	0.626	4.24	4.10	3.381	<0.001	4.13	5.37	3.91	4.14	<0.001	30.92	4.23	3.63
		1		37.62	31.77		34.01	32.74	33.02		36.37	39.70	36.89	29.38		47.48	42.31	30.04
		2		13.54	13.68		13.58	14.12	14.07		13.69	14.93	14.13	13.16		10.41	14.10	13.50
		3		28.54	33.10		31.35	32.09	32.70		30.04	26.03	30.44	34.20		6.47	25.91	34.32
		4		9.15	10.05		9.76	9.45	9.20		9.29	8.45	8.76	10.69		2.37	7.51	10.81
		>4		6.98	7.19		7.06	7.52	7.63		6.48	5.54	5.86	8.44		2.37	5.94	7.71
	linear whorls lineal	0	0.052	91.84	91.05	0.043	91.45	90.56	89.70	<0.001	91.73	92.84	92.39	90.31	<0.001	92.74	89.79	91.96
		1		4.21	4.99		4.60	5.76	5.82		4.73	3.28	3.60	5.33		3.943	4.26	4.92
		2		3.21	3.23		3.24	2.78	3.62		2.83	3.34	3.29	3.56		2.21	5.11	2.44
		>2		0.75	0.73		0.72	0.91	0.87		0.72	0.54	0.72	0.80		1.11	0.84	0.68
Body-neck	Circular whorls	0	0.222	4.78	4.58	<0.001	4.79	3.79	2.52	<0.001	4.87	4.28	5.51	4.25	<0.001	36.59	4.88	3.88
		1-2		7.58	7.57		7.61	7.31	7.08		8.34	6.94	8.84	6.56		9.15	7.28	7.67
		3-4		15.94	15.90		16.07	14.15	15.17		16.95	19.86	15.53	14.23		10.10	15.40	16.26
		5-6		20.28	20.10		20.28	18.82	19.65		20.36	20.58	21.28	19.55		13.09	19.79	20.47
		7-8		18.48	18.84		18.76	17.87	19.03		18.80	18.64	18.57	18.69		11.36	20.05	18.30
		9-10		13.83	13.83		13.66	15.67	14.94		13.33	12.93	13.39	14.64		9.46	14.29	13.73
		>10		19.10	19.18		18.83	22.38	21.62		17.36	16.78	16.88	22.09		10.25	18.31	19.70
	Linear whorls	0	<0.001	37.79	38.78	<0.001	38.94	34.39	31.53	<0.001	39.32	39.90	41.71	36.21	<0.001	51.42	37.64	38.47
		1-2		17.58	16.71		16.96	17.64	17.61		18.37	21.20	18.41	14.45		15.14	19.91	15.85
		3-4		11.91	12.70		12.29	13.00	14.94		11.88	10.42	10.86	13.81		11.67	12.58	12.35
		5-6		15.23	15.69		15.53	15.34	15.57		15.08	13.76	12.95	17.10		9.78	14.00	16.29
		7-8		10.53	9.90		10.06	10.94	10.38		9.43	9.59	9.774	11.04		5.52	8.55	10.90
		9-10		4.35	3.96		3.94	5.45	5.90		3.66	3.05	4.04	4.76		3.47	4.12	4.11
		>10		2.61	2.27		2.27	3.25	4.09		2.26	2.08	2.26	2.63		3.00	3.20	2.04
Limbs	Cir-cular	0	<0.001	88.02	90.11	0.004	89.40	87.85	91.35	<0.001	89.82	90.58	90.21	88.39	<0.001	94.80	85.57	90.84
		1		1.92	1.41		1.60	1.73	1.26		1.62	1.60	1.56	1.58		0.79	2.14	1.38

	2		9.37	8.06		8.49	9.72	7.31		8.01	7.31	7.79	9.54		4.26	11.63	7.32
	>2		0.68	0.42		0.52	0.71	0.08		0.56	0.51	0.44	0.49		0.16	0.66	0.46
Linear whorls	0		91.34	92.27		91.98	90.86	92.93		92.78	92.95	92.78	90.60		95.90	88.70	93.23
	1	<0.001	0.98	0.96	0.038	0.99	0.68	0.87	<0.001	0.99	0.94	0.92	0.96	<0.001	0.63	1.41	0.79
	2		7.28	6.49		6.70	8.06	6.13		5.91	5.79	5.97	8.08		3.47	9.42	5.73
	>2		0.41	0.27		0.32	0.41	0.08		0.32	0.31	0.33	0.32		12.00	0.48	0.26
